# The neuroscience of respect: insights from cross-cultural perspectives

**DOI:** 10.3389/fpsyg.2023.1259474

**Published:** 2023-12-19

**Authors:** Rabia Khalaila, Jayashree Dasgupta, Virginia Sturm

**Affiliations:** ^1^Memory and Aging Center, Global Brain Health Institute, San Francisco, CA, United States; ^2^Weill Institute for Neurosciences, University of California at San Francisco, San Francisco, CA, United States; ^3^Nursing Department, Zefat Academic College, Zefat, Israel; ^4^School of Psychology, Trinity College Dublin, Dublin, Ireland; ^5^Samvedna Care, Gurugram, India; ^6^John Douglas French Alzheimer's Foundation Endowed, Los Angeles, CA, United States

**Keywords:** cultural values, neuroscience, respect, neuroanatomical, neurodegenerative disorders

## Abstract

Cultural values such as respect influence cognition, emotion, and behavior by modulating brain functioning. This mini-review discusses the cultural differences of respect as an essential human value, and the neural underpinnings accompanying them. Although neuroscientific studies are limited, we outline potential brain structures and networks that contribute to respect and use clinical examples to illustrate how behavior changes when these neural systems fail. A better understanding of the neuroanatomical basis of respect and its neural manifestations across cultures will help to advance current conceptualizations of the biology of human values.

## Introduction

The human brain is biologically prepared to acquire culture (Fiske, 2002). By shaping our identities, perceptions, and behaviors, culture provides a framework for understanding and navigating the world around us and shapes our brain activity (Han and Humphreys, 2016). Culture is a complex concept that encompasses a myriad of practices including language, dress, food, and music. Shared values, beliefs, and behaviors are also central elements of culture that bond people together and create a common identity that sets them apart from other groups.

Respect is a fundamental human value that refers to the consideration and admiration that one shows for oneself, others, and the environment (Li and Fischer, 2007). As respect motivates people to validate and acknowledge the feelings of others, it has a crucial role in human relationships, one facet of which is empathy, the ability to feel and comprehend the experiences of others (Decety and Jackson, 2004). The association between respect and empathy is likely bidirectional; when individuals respect others, they are more likely to feel empathy for them, and when they feel respected, they may express their emotions more openly and honestly, and elicit greater empathy. Feelings of mutual respect, therefore, can lead to better emotional connection and understanding between individuals (Li and Fischer, 2007).

While the value of respect may be universal, its specific manifestations differ across cultures (Mackenzie and Wallace, 2011). Therefore, the way in which respect influences the brain in people across cultures may also vary. Cultural neuroscience is an interdisciplinary area of study that combines theories and methods from the fields of cultural psychology and neuroscience (Han and Northoff, 2009; Ames and Fiske, 2010; Chiao et al., 2013; Han et al., 2013; Kim and Sasaki, 2014). This approach explores how culture shapes brain functioning and, conversely, how neural processes influence cultural values, beliefs, and practices (Nisbett et al., 2001; Nisbett and Masuda, 2003; Chiao et al., 2010; Kitayama and Park, 2010; Park and Huang, 2010; Rule et al., 2013; Han and Humphreys, 2016). Although relatively little is known about how cultural differences concerning respect influence brain functioning (Li and Fischer, 2007), prior studies can enlighten our understanding of the neural basis of respect and help to elucidate its varying role in cognition, emotion, and behavior across cultures (Mesquita and Walker, 2003).

## Cross-cultural displays of respect

Cultures around the world hold the value of respect in high regard, but social norms that guide demonstrations of respect vary from one culture to the next (Mackenzie and Wallace, 2011). While many cultures encourage respect for elders and those of higher social status, collectivist and individualistic societies have notable differences in cultural norms surrounding respect (Ingersoll-Dayton and Saengtienchai, 1999). Here we describe some cultural practices to illustrate commonalities and differences in the practice of respect across cultures, but it is important to note that these are generalizations and that demonstrations of respect can also differ between individuals in the same culture.

In collectivist societies, respect is deeply ingrained in traditional customs and norms that guide social interactions. Collectivist societies tend to emphasize the wellbeing of the group over the needs and desires of individuals, and conveying respect is an integral part of verbal and non-verbal communication. In Asian cultures, people communicate respect and honor for their parents through filial piety practices such as bowing, using honorific titles, speaking politely, maintaining harmony, and avoiding actions that may cause their parents “loss of face” or embarrassment (Ingersoll-Dayton and Saengtienchai, 1999). Elders in Latin American cultures are also addressed with formal titles, and people maintain adequate personal space during social interactions to show deference. Using greetings and expressions of politeness, such as “please” and “thank you,” as well as appropriate body language and gestures, is crucial for demonstrating respect (Calzada et al., 2010). In African cultures, respect is also expressed through greetings and gestures as well as attentive listening. Respect is extended to all members of the community, irrespective of age or social status, but elders are highly revered and considered to be a source of wisdom, guidance, and experience. Younger individuals, therefore, are expected to obey the older members of the community (Idang, 2015). In Middle Eastern and Indian cultures, respect is often linked to hospitality, honor, and family and underlies one's sense of duty toward elders. Most widely spoken Indian languages, for example, have a system of honorifics that conveys the degree of familiarity and formality within relationships (Bhatt, 2012). Respect for guests, elders, and those in positions of authority is demonstrated by using formal titles (Qidwai et al., 2017; Memon et al., 2021).

Individualistic societies, in contrast, prioritize values, norms, and practices that promote personal autonomy and independence. In these societies, individuals are encouraged to express their unique identities and to pursue their own goals. Independence is highly valued, and personal achievements, skills, and talents are key priorities (Hsieh, 2011; Grossmann and Santos, 2016). People in individualistic societies are expected to take responsibility for their lives, and their success is often measured on an individual basis. As equality and the protection of personal choices and rights are central tenets of individualistic societies, fostering environments with fair treatment of all is of paramount importance. Unlike collectivist societies, which focus on larger communities, individualistic societies focus on the nuclear family and emphasize the importance of self-sufficiency within a smaller family unit. While Western countries including Australia, Canada, Germany, the Netherlands, and the United States (Grossmann and Santos, 2016) often prioritize principles of individualism, there are still expectations and standards for showing respect to others. For example, using good manners and respecting personal space, privacy, personal boundaries, and consent are highly valued in Western cultures regardless of a person's gender, race, ethnicity, religion, social status, or other characteristics (Hsieh, 2011).

## Neuroanatomical basis of respect

Respect is a multifaceted value and so, too, are its neuroanatomical underpinnings. While there are no specific brain structures solely dedicated to respect, certain neural regions and networks may have crucial roles in the cognitive, emotional, and behavioral processes that foster respect (Etkin et al., 2015). To show respect in social contexts, one must first know the pertinent rules to follow (Memon et al., 2021). In the brain, the anterior temporal lobes contain all types of semantic knowledge and may be important for storing semantic knowledge about respect. The left anterior temporal lobe holds information about verbal concepts and objects and, thus, might create associations among words, facts, and social rules that are relevant to respect (Joyal et al., 2017). Although research on the neural basis of respect is limited, one previous study found that the left anterior temporal lobe participates in determining the extent to which one feels respect and admiration for others in various situations (Nakatani et al., 2019). The right anterior temporal lobe, in contrast, is essential for representing non-verbal concepts and socio-emotional information. By helping people to understand others' voice prosody, bodily movement, and facial behavior, the right anterior temporal lobe is important for understanding emotions and social information which may foster respect in interpersonal contexts (Rosen et al., 2005; Rankin et al., 2006).

Respect invokes feelings of admiration and appreciation (Nakatani et al., 2019), and brain networks that support emotions are also likely important for this other–oriented value (Etkin et al., 2015; Nakatani et al., 2019; Sander and Nummenmaa, 2021). Through connections with the ventral striatum, amygdala, hypothalamus, and periaqueductal gray, the anterior cingulate cortex and ventral anterior insula are critical for generating and sensing internal changes in the body that arise during emotions, empathy, and reward (Seeley et al., 2012; Vogt, 2014; Etkin et al., 2015). Working together, this system allows people to detect salient information in the environment, to produce and experience emotions, and to nurture feelings of social connection (Decety, 2015). These regions may also contribute to the positive feelings that arise as people value the worth and dignity of others and admire their skills or virtues (Immordino-Yang et al., 2009). Feeling respected by others may elicit similar warm feelings. With close connections to admiration, gratitude, and elevation—prosocial emotions that people feel when witnessing others' exemplary behavior (Algoe, 2009)—respect may foster mutually enjoyable experiences (Algoe, 2019).

One must know the social rules that guide respectful behavior, but interpersonal interactions are dynamic, and social rules can change in an instant. In South Korean culture, for example, humor is often a part of social interaction, but what one person finds funny, another might find offensive. Understanding the appropriateness of humor requires constant awareness of the current context and the people involved. A joke that is acceptable in one setting may be inappropriate in another, and what was considered funny yesterday may not be perceived the same way today (Kim and Plester, 2019). To ensure that one acts in a respectful manner, one must track ongoing situations and adjust behavior as needed. The successful navigation of complex social situations, therefore, requires flexibility in cognition and behavior. The orbitofrontal cortex is an area in the ventral frontal lobes that guides decision-making and helps people to modify their actions and adapt to changing social environments (Kringelbach and Rolls, 2004). By allowing people to monitor and adjust their thoughts, actions, and emotions to each dynamic context, the orbitofrontal cortex is critical for fostering displays of respect during ongoing social interactions.

## Brain damage can disrupt respect

Neuroimaging studies of healthy individuals can elucidate the neural networks that promote respect, but clinical studies have revealed how respect can decline when there is dysfunction in these brain systems. Perhaps the most well-known person who lost respect for social norms was Phineas Gage, a railroad worker who suffered a terrible injury to his orbitofrontal cortex when an iron rod shot through his skull and brain. Although Gage survived this horrific accident, his behavior after brain injury altered radically. Prior to the accident, he had been a well-respected man who adhered to typical social norms. But after his orbitofrontal cortex injury, Gage began to swear and drink to excess. He was no longer the man he had been, and his social interactions were often problematic as he could no longer control his behavior or show respect for others (O'Driscoll and Leach, 1998).

In certain neurodegenerative disorders, respectful behavior also declines when atrophy progresses through the orbitofrontal cortex and connected neural networks. The behavioral variant of frontotemporal dementia (bvFTD) is a neurodegenerative disorder in which there is selective tissue loss in the orbitofrontal cortex as well as the ventral anterior insula, anterior cingulate cortex, amygdala, and anterior temporal lobes (Seeley et al., 2012). In bvFTD, changes in social behavior, personality, and emotion (e.g., apathy, loss of empathy, and compulsivity) are common (Rosen et al., 2005; Rankin et al., 2006; Sturm et al., 2006, 2016). As people with bvFTD may defy social norms and hurt other people's feelings, their behavior may also be considered disrespectful. Atrophy in the orbitofrontal cortex and connected brain networks, may contribute to loss of respect in bvFTD because patients are no longer able to abide by the social rules that are necessary in showing respect.

## Cultural influences on brain functioning

Although much remains unknown about how culture shapes brain functioning, a growing body of research suggests that activity patterns in the brain allow people to think and behave in culturally appropriate ways (Nisbett et al., 2001; Domínguez et al., 2009; Kitayama and Park, 2010; Park and Huang, 2010; Rule et al., 2013; Han and Humphreys, 2016). By repeatedly engaging in cultural perspectives and practices, people may shape their own brain network in specific ways that allow them to think and act according to cultural norms without much deliberation or effort (Kitayama and Uskul, 2011). How a culture influences brain functioning may vary, however. People from collectivist societies, for example, focus more on context and relationships and tend toward a more holistic (interdependent) cognitive style that is characterized by thematic categorization of objects, a focus on contextual information and relationships in visual attention, an emphasis on situational causes in attribution, and dialecticism (Nisbett et al., 2001; Varnum et al., 2010; Han and Humphreys, 2016). People from individualistic societies, in contrast, focus more on objects and attributes and tend toward a more analytic (independent) cognitive style that is characterized by taxonomic and rule-based categorization of objects, a narrow focus on visual attention, and the use of formal logic in reasoning (Nisbett et al., 2001). Consistent with these differences, people from Western cultures tend to remember more details of objects and events in autobiographical memory than people from Eastern cultures (Wang, 2001).

Neuroimaging studies have also found distinct patterns of neural activation in people from different cultures (Rule et al., 2013; Zhang et al., 2022). One study compared brain activity in participants from the United States and Japan in response to affectively laden stimuli and found cultural differences in reward system activity (Freeman et al., 2009). While in the scanner, participants viewed images that depicted dominance (e.g., power, control, and authority) or subordination (e.g., inferiority, submissiveness, or feeling being controlled by others). While the American participants—who are encouraged to be independent, assertive, and skeptical of authority—showed increased activity in the medial prefrontal cortex and caudate to the stimuli that elicited feelings of dominance, the Japanese participants—who are encouraged to be deferent, cooperative, and mindful of their social obligations—activated those same areas in response to the stimuli that elicited feelings of subordination (Freeman et al., 2009). These results suggested that culturally-preferred social information elicited greater activity in reward systems in each group.

In another study, European Americans and East Asians completed simple visuospatial tasks that required them to make absolute judgments (ignoring visual context) or relative judgments (taking visual context into account) while in the scanner (Hedden et al., 2008). As European Americans tend to make absolute judgments, and East Asians tend to make relative judgments, the researchers hypothesized that overriding these culturally-based tendencies would require greater effort and cognitive control. Consistent with their expectations, the results showed that activity in frontoparietal regions that support attention and cognitive control was greater when the participants in each group made judgments that were not culturally preferred (Hedden et al., 2008). Other studies have found these cultural differences in neural activity also extend to other areas of cognition including visual perception (Gutchess et al., 2006; Goh et al., 2010; Rule et al., 2013), causal attribution (Han et al., 2011), mental calculation (Tang et al., 2006), and self-reflection (Zhu et al., 2007).

## Respect and cultural neuroscience

Cultural practices and values can modulate brain functioning and influence how people process social, emotional, and cognitive information (Etkin et al., 2015; Nakatani et al., 2019; Sander and Nummenmaa, 2021). Much remains unknown, however, about how specific values such as respect, shape neural processes in various cultures. Although it is an important area of research, there are many factors that make the neuroscience of respect a challenging topic to study. Social norms and expectations surrounding respect are not fixed or static but change and evolve across time, people, and situations. As technological advancements and globalization continue to develop, intercultural interactions and social norms also continue to change. With an increasingly connected world, individuals may inhabit multiple cultures and hold multiple cultural identities (Nisbett et al., 2001). Their ability to shift flexibly between different modes of thinking and acting may make it difficult to determine which cultural values shape brain functioning in a given setting. A more sophisticated conceptualization of the neural underpinnings of respect will require the identification of common brain networks that support respectful behavior across cultures and differences in brain activity that contribute to culturally-specific manifestations of respect.

## Conclusions

The neuroscience of respect is a complex and evolving field that remains replete with unanswered questions. By integrating the study of culture and neuroscience, however, we can gain important insights into the complex interplay between human values and the brain. A better understanding of respect has the potential to promote cross-cultural understanding and foster more inclusive and harmonious societies. The value of respect is already recognized as a core value in diversity, equity, and inclusion frameworks across sectors (Kiradoo, 2022), but leaders and co-workers from different cultures may communicate about or process information differently, as detailed in this review. More research is needed to elucidate how knowledge about the biological basis of respect can inform interventions and strategies that promote respectful behavior and reduce conflict in diverse cultural settings.

## Author contributions

RK: Conceptualization, Project administration, Writing–original draft. JD: Conceptualization, Writing–original draft. VS: Supervision, Writing–review & editing.
